# Effect of continuous lily cropping on rhizosphere microbial structures

**DOI:** 10.3389/fmicb.2025.1658893

**Published:** 2025-10-27

**Authors:** Yan Zhang, Juan Wang, Yan Zhao, Jie Liu

**Affiliations:** Provincial Engineering Research Center of Lily Germplasm Resource, Hunan University of Technology, Zhuzhou, Hunan, China

**Keywords:** lily, soil, microbiology, continuous cropping, microbial community structure

## Abstract

Lily is a highly valued economic crop in China, possessing both significant ornamental and medicinal value. However, the phenomenon of farmers repeatedly cultivating lilies in the same area is quite serious, leading to soil nutrient deficiency and a decline in yield and quality. This study focused on the soil of lily continuous cropping as the research object. By analyzing the changes in the physicochemical properties and microbial community structure of lily soil over the same continuous cropping years, the effects of continuous cropping on lily soil were explored. The results show that with the increase of continuous cropping years in lily fields, soil acidification intensifies; the contents of organic matter, nitrogen, phosphorus, and potassium all decrease; the activities of sucrase and acid phosphatase decline; and the activities of catalase, protease, and urease increase. The results revealed the diversity of microbial structures in the rhizosphere soil of the lily “Longya,” providing a theoretical basis for exploring the rules of microbial community change, screening and utilizing beneficial microorganisms, and maintaining the growth and development of lilies.

## 1 Introduction

Continuous cropping, where the same crop is growing on the same land for years (Alami et al., 2021), is a common farming practice in China. It is widely found in the cultivation of fruit trees, vegetables, flowers, and Chinese medicinal herbs. However, continuous cropping often leads to continuous cropping obstacles (CCO), such as soil acidification, soil nutrient deficiency, imbalance in the soil microbial community, and intensification of crop diseases, which seriously affect crop yield and quality, causing economic losses and restricting industry development. Studies have shown that continuous cropping can severely aggravate ginseng root rot, leading to fibrous root shedding, plant death, and yield reductions ranging from 80 to 100% (Li and Jiang, 2018). Similarly, in the case of *Fagopyrum tararicum*, yields decreased by 6.36, 24.85, 78.62, and 83.10% over four consecutive years of monoculture, resulting in substantial economic losses (Zhang et al., 2021). As the number of continuous cropping years increases, key indicators such as soil nutrient availability, soluble protein content, soil enzyme activity, total leaf chlorophyll content, and the abundance of actinomycetes gradually decline (Alami et al., 2021).

The Longya lily (*Lilium brownii* var. *viridulum*), one of China’s main edible lilies, is an excellent plant resource that severs as both a medicine and food source. Its bulbs are rich in sugar, protein, minerals, alkaloids, and steroid saponins and other effective ingredients, with the effects of removing heat, easing thirst, moistening lungs, and soothing the nerves. It has a large demand and high economic value in the traditional Chinese medicinal materials and high-end agricultural products markets. As a perennial bulbous herb, the lily is also facing the same as traditional crops, resulting in an estimated 50 million dollars lost for farmers in Gansu Province (Shi et al., 2020).

Changes in a soil microbial community are regarded as one of the main factors of CCO (Larkin, 2008). Many reports have revealed that microbial composition is closely related to the transformation and utilization of soil nutrients, which directly affects crop growth, and discussed some important bacterial and fungal communities related to continuous cropping obstacles. The results of eggplant (Xiao et al., 2024), industrial hemp (Guo et al., 2022), and cucumber (Sun et al., 2021) showed that continuous cropping would change soil enzyme activity and physicochemical properties. Soil bacterial diversity and richness index decreased, beneficial microorganisms decreased and harmful microorganisms increased, resulting in a shift in the rhizosphere microenvironment from the “bacterial type” to the “fungal type.” Several studies believed a significant decline in the abundance of beneficial microorganisms with prolonged tobacco cropping (Yu et al., 2024), while Ding et al. (2024) found the relative abundance of bacterial genera associated with nutrient cycling (e.g., Sphingomonas) increased while potential plant pathogenic fungi and beneficial microorganisms showed synergistic increases with the duration of continuous cropping. The primary CCO factors vary among different medicinal plants. Liu et al. (2021) reported that a decrease in soil pH with increasing Continuous cropping years (CCYs) was the main factor causing CCOs in American ginseng. *Boehmeria nivea* (L.) Gaudich concluedes that an increase in the diversity of soil bacterial microorganisms and a decrease in Actinomycetes diversity with increasing CCYs are key factors driving CCOs (Wang et al., 2020) suggested that the decrease of fungal diversity and richness can trigger the pathological condition of lisianthus plants (Zhang et al., 2024). The underlying factors are complex, and the factors causing soil degradation and their changing trends over continuous cropping years are differ significantly due to variations in experimental areas, crop types, soil types, and daily field management.

There is limited research on the changes in the soil microbial community during different CCYs of the lily. The loss of fungal diversity, simplification of fungal community structure, accumulation of pathogenic fungal genera, and depletion of beneficial fungal genera contribute to the negative effects of replanting in Lanzhou lily (Yang et al., 2023). After continuous cropping, soil bacterial microbial community diversity significantly increased, while fungal microbial diversity decreased in lilies planted in the red soil of Yunnan Province. Moreover, the increase of *Fusarium* was considered to be one of the main reasons for the CCO (Li et al., 2022). However, at present, research on the continuous cropping obstacles of Longya lily mainly focuses on ornamental varieties (such as Asian lily and Oriental lily), while the mechanism of continuous cropping obstacles of medicinal lilies (especially Longya lily) lacks systematic analysis, the reasons for the changes in the structure and composition of the microbial community during continuous cropping of Longya lily are not clear, and further in-depth studies are needed. In this study, we collected data on soil physicochemical properties, enzymatic activity, and microbial communities of different continuous cropping years. The objectives of the study were: (1) to investigate changes in microbial communities and diversity after different durations of continuous cropping; (2) to identify the time when soil physicochemical properties deteriorate after continuous cropping; (3) to reveal the relationship between microorganism and environmental factors; and (4) to identify the key microorganisms that make continuous cropping problematic. This helps clarify the key factors causing specific CCO of Lilium longuera, reveals the mechanism of soil microbial community imbalance, provides theoretical support for optimizing soil management and solving the CCO of Lilium longuera, and is conducive to the sustainable development of the Lilium industry.

## 2 Materials and methods

### 2.1 Field site description and soil sampling collection

The fields were located in the Lukou Lily Plantation, at Zhuzhou City, Hunan Province, China (113°1′E, 27°31’N). It has a typical subtropical monsoon climate, with an annual average precipitation of 1,500 mm, an average temperature of 17 °C, and a frost-free period of 286 days. The lily variety used in the experiment was Lonya lily (*Lilium brownii* var. *viridulum*), and all lily plantations used the same agricultural management practices. Four treatments were designed: L1 (continuous cropping 1 year), L2 (continuous cropping 2 years), L3 (continuous cropping 3 years), and L4 (continuous cropping 4 years). Soil samples were collected for each treatment using a five-point sampling method, and each treatment consisted of three replicates. Each sample is selected with an area of 1 m × 1 m. Before sampling, use a sampling shovel to remove the weeds and stones on the surface of the soil. Then, dig a soil sample of 10 to 20 cm and place it in a tray. Remove small stones, fine roots, insects, and other debris from the soil sample. Use the four-part method to pour the soil sample into a sterile bag and place it in an ice box. Then it is transported to a − 80 °C refrigerator in the laboratory. Another part of the soil samples was sieved through a 2 mm mesh sieve and then dried at 60 °C for analysis of soil physicochemical properties, or stored in a − 20 °C refrigerator for enzyme activity detection.

### 2.2 Soil properties determination

All of the soil chemical property analyses were based on Bao Shidan and Guan Songyin’s methods. Soil pH (soil:water = 1:2.5, w/v) was determined using a pH meter with a glass electrode. Alkaliolytic nitrogen (AN) was determined by the alkaliolytic diffusion method. Total nitrogen (TN) was determined by the Kjeldahl method. Extraction with NaHCO_3_ was used to determine available phosphorus (AP) and total phosphorus (TP) according to the molybdenum blue colorimetric method. Soil organic matter (SOM) was analyzed by potassium dichromate volumetric method. Total potassium (TK) was fused with NaOH and determined by flame spectrophotometry. Available potassium (AK) was extracted by NH_4_OAc and subjected to flame photometry.

Urease activity was determined by the sodium phenol-sodium hypochlorite colorimetric method. Urease catalyzes the hydrolysis of urea to produce ammonia and CO₂. Ammonia reacts with sodium phenolate and sodium hypochlorite under alkaline conditions to form blue indophenol. The absorbance is measured at 625 nm, and the enzyme activity is calculated. Protease activity was determined by ninhydrin colorimetry. Protease hydrolyzes protein to produce amino acids. Amino acids react with ninhydrin under heating conditions to form a purple compound. The absorbance is measured at 570 nm, and the enzyme activity is calculated. The activity of Sucrase was determined by 3, 5-dinitrosalicylic acid colorimetry. Sucrase hydrolyzes sucrose to form reducing sugar. Under alkaline conditions, the reducing sugar reacts with DNS to form a red-brown amino compound. The absorbance is measured at 540 nm to calculate the enzyme activity. Acid Phosphatase activity was determined by the colorimetric method. Acid phosphatase hydrolyzes disodium phosphate to produce phenol. Phenol reacts with 4-aminoantipyrine and potassium ferricyanide under alkaline conditions to form a red quinone compound. The absorbance is measured at 510 nm, and the enzyme activity is calculated.

Enzyme activity= $\frac{C\times V}{W\times T}$ .

C: Concentration obtained from the standard curve (μg/mL).

V: Total volume of the reaction solution (mL).

W: Sample mass (g).

T: Reaction Time (h).

Catalase activity was determined by potassium permanganate titration. Catalase decomposes H₂O₂, and the remaining H₂O₂ reacts with potassium permanganate under acidic conditions. The enzyme activity is calculated by titrating the amount of potassium permanganate consumed.

Catalase activity= $\frac{V_{\text{blank}}-V_{\text{sample}}}{W\times T}$ .

V: KMnO₄ consumption (mL).

W: Sample mass (g).

T: Reaction Time (min).

### 2.3 High-throughput sequencing analysis

Soil genomic DNA was extracted using the Soil FastDNA Kit (MP Biomedical, Illkirch, France) in accordance with the instructions. The quality of the extracted DNA was detected and analyzed by agarose gel electrophoresis, and then common primers were used:

Positive: 341F (5 ‘-CCTAYGGGRBGCASCAG-3’).

Reverse: 806R (5´-GGACTACNNGGGTATCTAAT-3′) amplifiers the V3-V4 hypervariable region of bacterial 16S rRNA.

Use universal primers:

Positive: ITS1F (5’-CTTGGTCATTTAGAGGAAGTAA-3′).

Reverse: ITS4R (5’-TCCTCCGCTTATTGATATGC-3′) was used for PCR amplification of the fungal ITS RNA gene sequence.

The PCR products were detected by 2% agarose gel electrophoresis. The thermal cycling conditions for PCR amplification are as follows: initial denaturation at 95 °C for 3 min, followed by 30 cycles of denaturation at 95 °C for 30 s, annealing at 55 °C for 30 s, and extension at 72 °C for 30 s. Library construction was performed using the NEBNext® Ultra™ IIDNA Library Prep Kit. The constructed library was quantified using the Qubit dsDNA HS detection kit and Qubit 4.0 fluorometer (Invitrogen, Thermo Fisher Scientific, Waltham, MA, USA). After the library is qualified, on-machine sequencing will be performed using Illumina NovaSeq6000.

### 2.4 Statistical analysis

The data were analysed by one-way ANOVA followed by Duncan’s multiple range tests using the SPSS Statistical Software Package ver. 20.0 (SPSS Inc., Chicago, IL, USA) to evaluate the significant changes in soil index parameters between different years. Differences at the *p* ≤ 0.05 level were considered statistically significant. Drawings were created using PRISM (version 7.0) and Mothur (version 1.17.0). The OTUs that reached a 97% nucleotide similarity level were used for alpha diversity (Shannon) and richness (Chao1), and Spearman’s correlation heatmap was generated based on the relative abundance of OTUs using the R Package (ver. 2.15; The R Project for Statistical Computing, http://www.R-project.org). A heatmap was generated using the heatmap package of the R tool to explore the changes in soil microbial abundance. To compare the common or unique microbial species in different regions, we used the R package to create OTU-level Venn diagrams. Principal component analysis (PCA) was performed using OTUs for each sample in the Mothu. Three alpha diversity indices, the number of OTUs, the Shannon index, and the Chao1 estimator of richness, and were calculated in QIIIME (Caporaso et al., 2010). The microbial communities were compared by Bray-Curtis distance similarities based on the abundance of OTUs. Nonmetric multidimensional scaling (NMDS) based on the Bray–Curtis similarity matrix was visualized using the R package vegan. LEfSe, allowing researchers to identify both statistical significance and biological relevance, was performed on the website.^1^ The taxa from phyla to genera with absolute LDA scores over 3 and *p*-values less than 0.05 are shown. The relationship between environmental factors and phylum diversity was analyzed using redundancy analysis (RDA) with Canoco for Windows (ver. 4.5). The scaling of RDA is a Correlation Biplot.

## 3 Results

### 3.1 Soil chemical properties

Table 1 shows a gradual decrease in soil pH and nutrients, such as organic matter content, available nitrogen, available phosphorus, and available potassium, with the increase in continuous cropping years. The soil pH ranged from 5.07 ~ 5.67 and was ordered L1 > L2 > L3 > L4. Compared to L1, the SOM decreased by 26.50%, TN decreased by 33.54%, AN decreased by 25.71%, AP decreased by 63.57%, TK decreased by 27.07%, and AK decreased by 65.51% in L4. The pH, AP, TK, and AK differed significantly between L1 and L2. The soil nutrients in L3 had significantly declined compared with L2. No significant difference was found in TP content as the duration of continuous cropping increased.

**Table 1:** Soil physicochemical properties under different continuous cropping years.

No.	pH	SOM (g kg^−1^)	TN (g kg^−1^)	AN (mg kg^−1^)	TP (g kg^−1^)	AP (mg kg^−1^)	TK (g kg^−1^)	AK (mg kg^−1^)
L1	5.67 ± 0.08a	21.95 ± 0.71a	1.61 ± 0.13a	120.43 ± 4.84a	1.26 ± 0.11a	18.96 ± 1.24a	26.97 ± 1.05a	734.40 ± 11.62a
L2	5.31 ± 0.06b	21.33 ± 0.58a	1.39 ± 0.06a	111.40 ± 3.64a	0.96 ± 0.17a	11.37 ± 1.24b	23.81 ± 0.81b	533.72 ± 14.07b
L3	5.16 ± 0.02bc	18.89 ± 0.68b	1.09 ± 0.04b	95.65 ± 2.81b	0.73 ± 0.01a	8.66 ± 0.60bc	21.90 ± 0.41bc	262.75 ± 4.20c
L4	5.07 ± 0.03c	16.13 ± 0.92c	1.07 ± 0.01b	89.47 ± 4.51b	0.95 ± 0.03a	6.91 ± 0.28c	19.67 ± 0.83c	253.33 ± 3.79c

L1, continuous cropping 1 year; L2, continuous cropping 2 years; L3, continuous cropping 3 years; L4, continuous c 4 years; SOM, soil organic matter; TN, total nitrogen; AN, available nitrogen; TP, total phosphorus; AP, available phosphorus total potassium; AK, available potassium.

Data are mean ± SD (*n* = 3). Different letters in the same column indicate significant differences at the *p* < 0.05 level.

### 3.2 Soil enzyme activities

**Figure 1:**
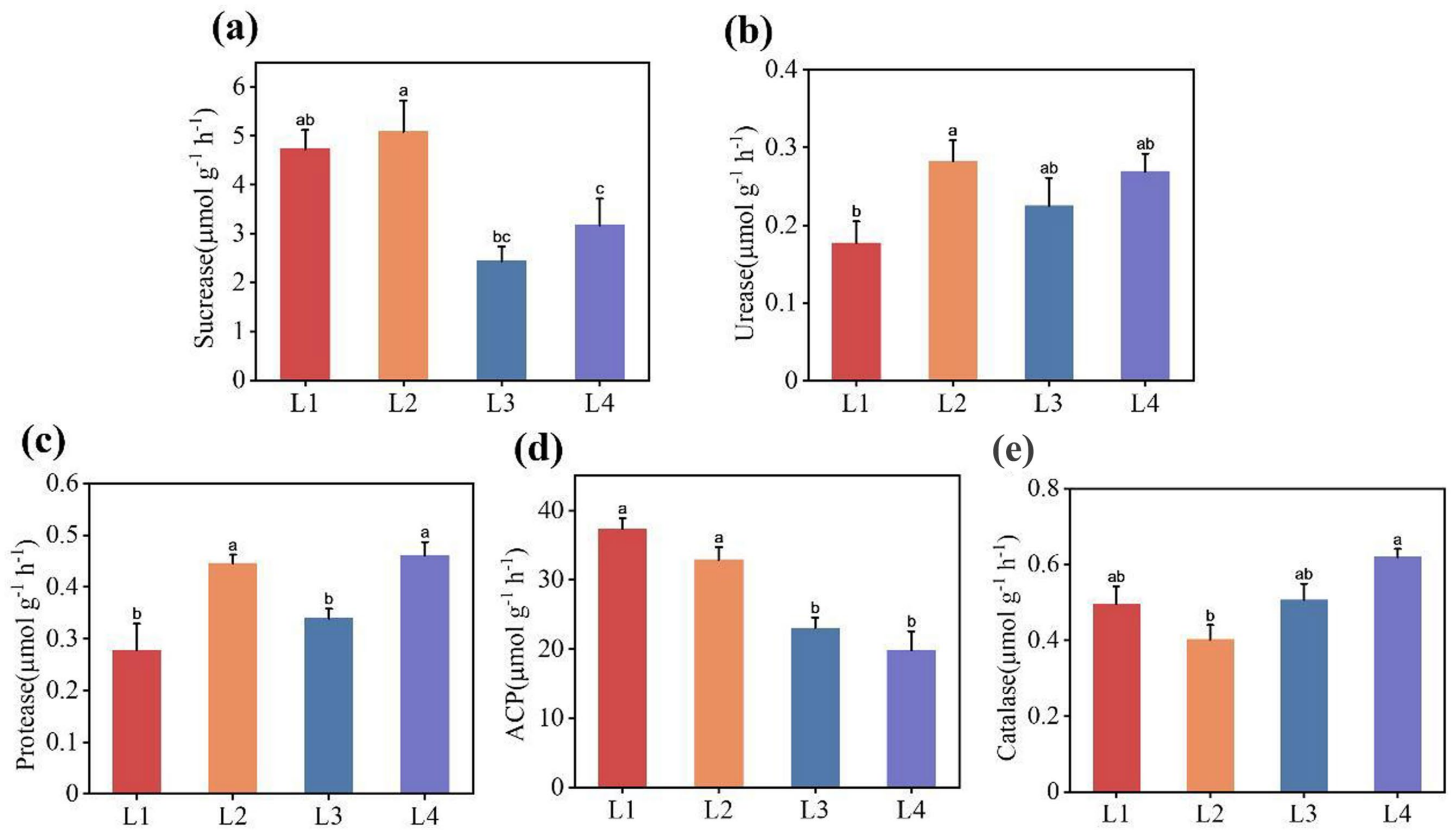
Soil enzymatic activities under different continuous cropping years **(a)** Sucrase activity, **(b)** Urease activity, **(c)** Protease activity, **(d)** Acid phosphatase activity, **(e)** Catalase activity. L1, continuous cropping 1 year; L2, continuous cropping 2 years; L3, continuous cropping 3 years; L4, continuous cropping 4 years. Different letters indicate significant differences among different durations of continuous lily (*p* < 0.05).

Five enzymatic activities of the soils under different continuous cropping years were compared and analyzed, as shown in Figure 1. With the increase of continuous cropping years, the sucrase activity and acid phosphatase activity showed a decreasing trend, whereas the urease activity and protease activity exhibited a gradual increasing trend and were higher in L2. The catalase activity initially dropped in L2 and then gradually increased in L3. When comparing L1 and L2, we observed a notable difference, with urease activity and protease activity being significantly higher in L1. In contrast, the acid phosphatase activity was considerably lower. The enzymatic activities in L3 were significantly decreased compared with those in L2.

### 3.3 Soil bacterial and fungal diversities

Alpha diversity was assessed using the ACE, Chao1, Shannon, and Simpson indexes to evaluate microbial richness and diversity (Table 2). The coverage of all soil samples was above 0.99 in the study, which meant the sequencing data was valid. The results revealed that continuous cropping affected the diversity of soil bacterial and fungal communities. The ACE, Chao 1, and Shannon indices for bacteria communities showed decreases with the increase of continuous cropping years, while the Simpson index decreased with the increase of continuous cropping years. Continuous cropping 3 years (L3) significantly reduced the soil bacterial ACE index, Chao 1 index, and Shannon index by 20.82, 20.87, and 6.66% respectively, compared with L1, while the Simpson index increased significantly by 52.78%. The ACE and Chao 1 indices of soil fungi showed a pattern of L2 > L3 > L1 > L4. The differences between L1 and L4 were significant, and no significant differences were found between L2 and L3. The fungal Shannon index was significantly lower in L1 than in the others, with a pattern of L2 > L3 > L4 > L1 and no significant difference among L2, L3, and L4. The Simpson index of L1 fungi was significantly higher than that of others, with a pattern of L1 > L4 > L2 > L3.

**Table 2:** Soil bacterial and fungal alpha diversity in different continuous cropping years.

Microorganisms	Cropping year	ACE	Chao 1	Shannon	Simpson	Coverage
Bacteria	L1	3395.10 ± 334.46a	3363.85 ± 335.09a	7.21 ± 0.15a	0.0017 ± 0.003c	0.9977 ± 0.002a
	L2	2787.67 ± 142.18ab	2767.93 ± 146.51ab	6.99 ± 0.09a	0.0022 ± 0.003c	0.9984 ± 0.004a
	L3	2688.16 ± 140.11b	2661.77 ± 142.12b	6.73 ± 0.05bc	0.0036 ± 0.003b	0.9980 ± 0.005a
	L4	2365.86 ± 108.47b	2348.45 ± 109.27b	6.50 ± 0.05c	0.0050 ± 0.005a	0.9985 ± 0.009a
Fungi	L1	472.52 ± 77.58ab	468.30 ± 78.44ab	3.33 ± 0.53b	0.130 ± 0.054a	0.9994 ± 0.001b
	L2	576.14 ± 14.62a	574.24 ± 14.31a	4.00 ± 0.18a	0.0631 ± 0.016b	0.9997 ± 0.001a
	L3	508.80 ± 99.38a	505.83 ± 99.22a	3.92 ± 0.40a	0.055 ± 0.019b	0.9995 ± 0.001ab
	L4	458.24 ± 37.30b	456.91 ± 36.70b	3.63 ± 0.18a	0.079 ± 0.022b	0.9998 ± 0.001a

The different letters in the same column indicate significant differences at the *p* < 0.05 level.

L1, continuous cropping 1 year; L2, continuous cropping 2 years; L3, continuous cropping 3 years; L4, continuous cropping 4 years.

**Figure 2:**
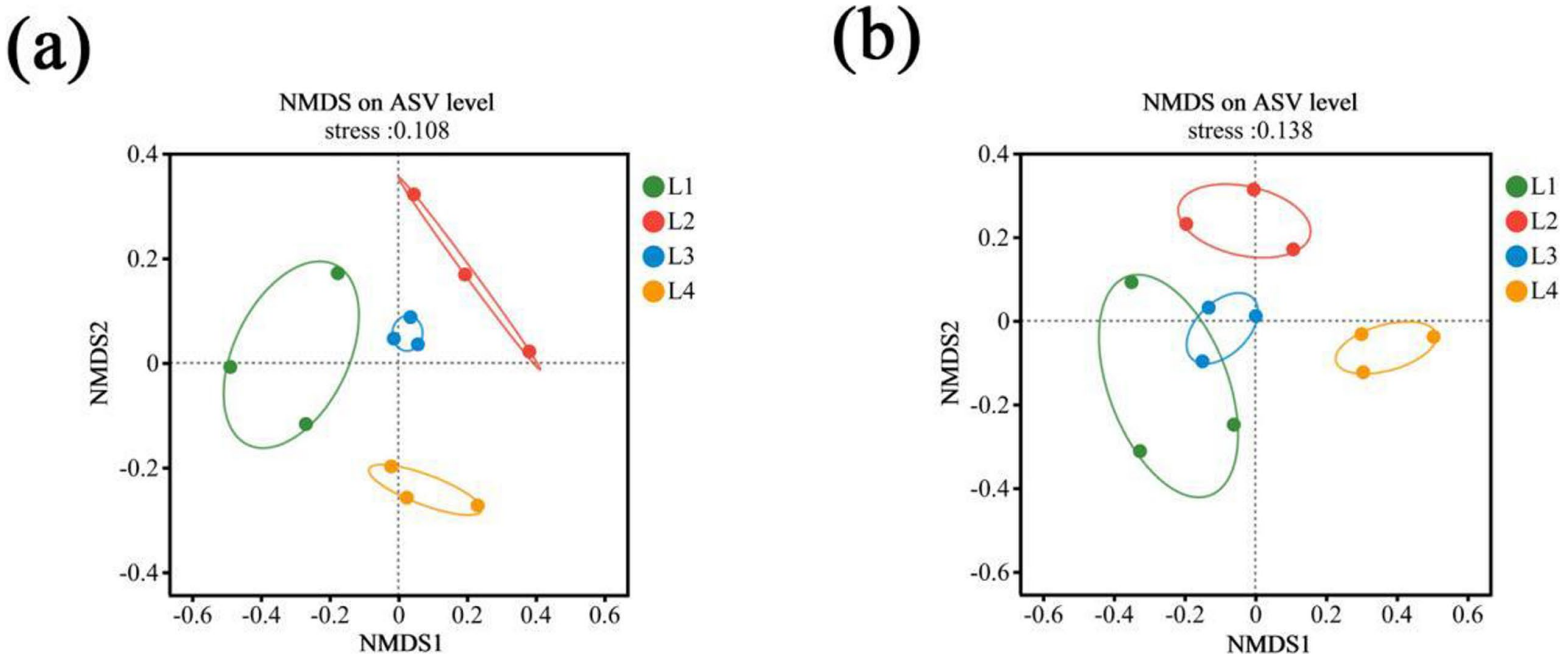
Non-metric multidimensional scaling (NMDS) analysis of bacterial **(a)** and fungal **(b)** communities in different continuous cropping years. L1, continuous cropping 1 year; L2, continuous cropping 2 years; L3, continuous cropping 3 years; L4, continuous cropping 4 years. Dots with different colors represent different years, and three repeats in each treatment.

Beta diversity was used to reflect the diversity differences of different continuous cropping years. The NMDS of soil bacteria, based on the Bray-Curtis distance, showed that L2 and L3 were clustered together in the same quadrant, suggesting a high similarity in bacterial community structure (Figure 2a). L4 was far away from others, indicating low similarity with others. Fungal communities under different cropping years were mainly separated along axis 1 (Figure 2b). The L1, L2, and L3 was located on the left side, while L4 were distributed on the right side, which displayed distinct differences in the soil fungal community structures between L1, L2, L3, and L4.

### 3.4 Soil microbial community composition and structure analysis

**Figure 3:**
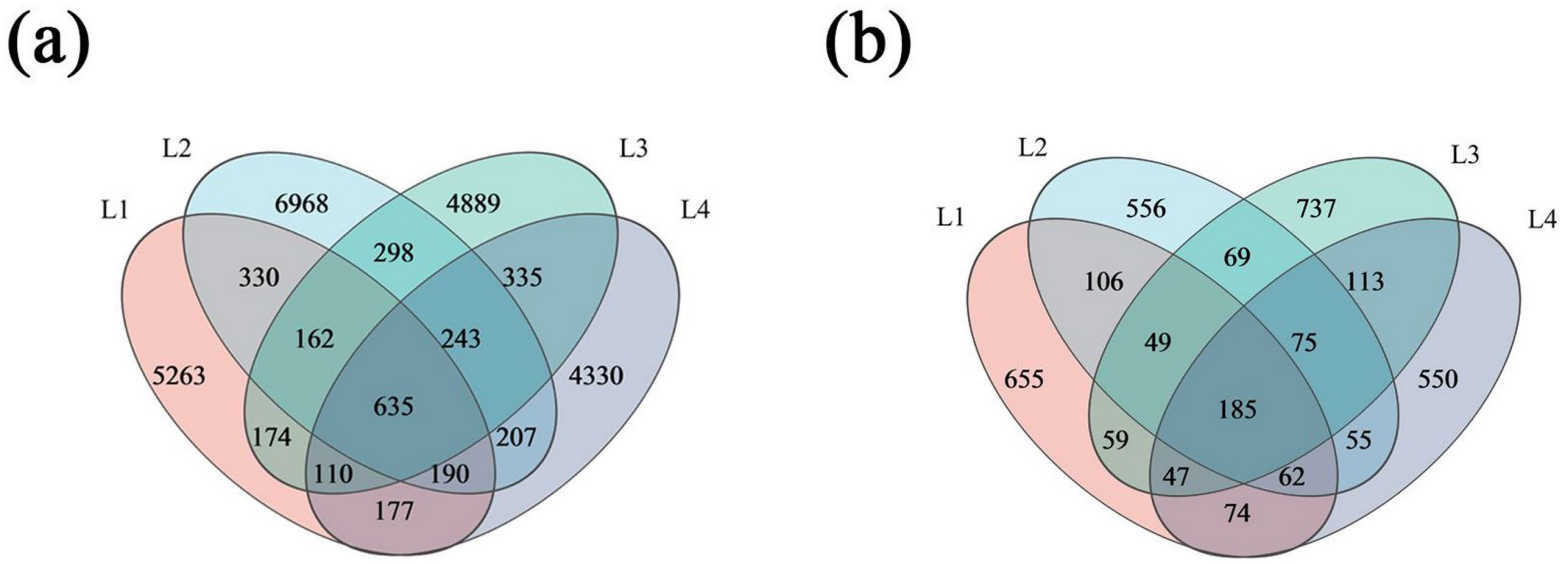
Venn diagram of bacteria **(a)** and fungi **(b)** in different continuous cropping years.

According to the Venn diagram in Figure 3a, the number of bacterial OTUs in the soil of the lily varied across different years, with the highest number of OTUs observed in L2, at 9033. There were 635 bacterial OTUs shared in different years. The number of unique bacterial OTUs in L1, L2, L3, and L4 were 5,263, 6,968, 6,846, and 4,330, accounting for 18.06, 23.91, 16.77, and 14.86% of the total OTUs, respectively. The Venn diagram in Figure 3b shows that the number of fungi OTUs was 1,237, 1,157, 1,334, and 1,161 in L1, L2, L3, and L4, with the highest number of OTUs in L3. The number of shared fungal OTUs was 185. The number of unique bacterial OTUs in L1, L2, L3, and L4 was 655, 556, 737, and 550, respectively, accounting for 13.40, 11.37, 15.07, and 11.25% of the total OTUs, respectively.

**Figure 4:**
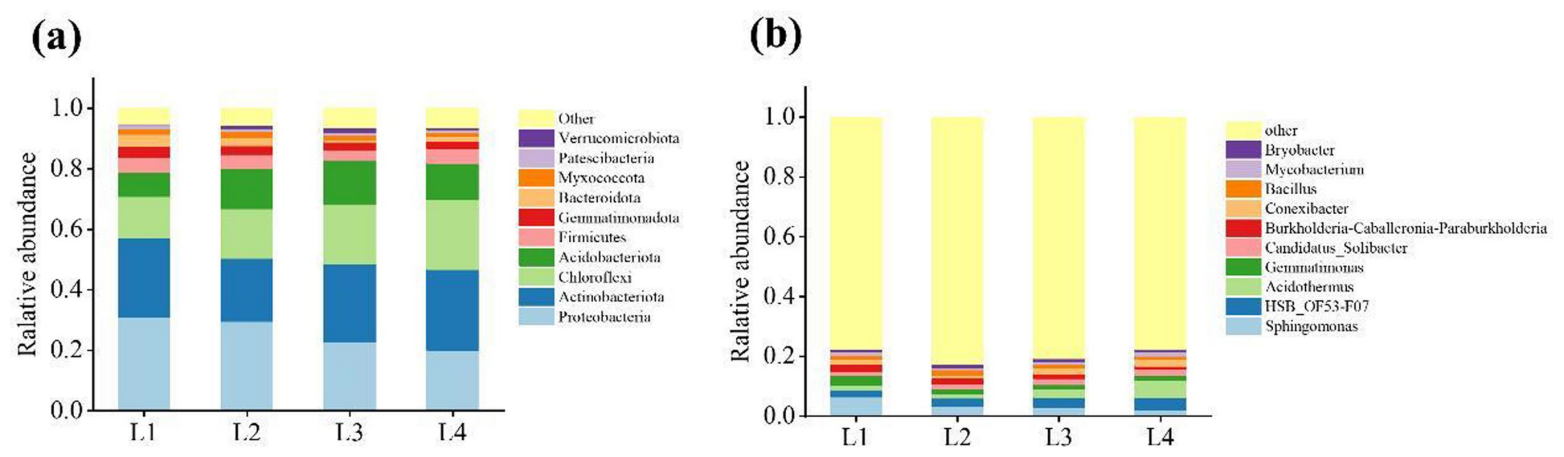
The distribution of dominant bacteria in different continuous cropping years at phyla level **(a)** and genus level **(b)**.

The dominant bacterial phylum in different continuous cropping years were Proteobacteria (20.05–30.81% of total relative abundance) and Actinobacteriota (20.89–26.62%), followed by Chloroflexi (13.59–23.05%), Acidobacteriota (8.03 -14.44%), Firmicutes (3.27–4.90%), Gemmatimonadota (2.30–3.82%), Myxococcota (1.26–1.90%) (Figure 4a). With the increase of continuous cropping years, the abundances of Proteobacteria and Gemmatimonadota in soil decreased by 34.92 and 39.83%, respectively, while the abundances of Chloromyces and Acidobacteria increased by 41.03 and 33.35%, respectively. Figure 4b indicated that Sphingomonas (2.01% ~ 6.37%), Acidothermus (1.38% ~ 5.88%), Gemmatimonas (1.53% ~ 3.29%), Conexibacter (1.01% ~ 2.20%), and Bacillus (1.06% ~ 1.68%) were the dominant classes. The abundances of Sphingomonas, Gemmatimonas, Bacillus were reduced by 68.49, 53.37, 15.52%, and Acidothermus increased by 76.50% compared to L1.

**Figure 5:**
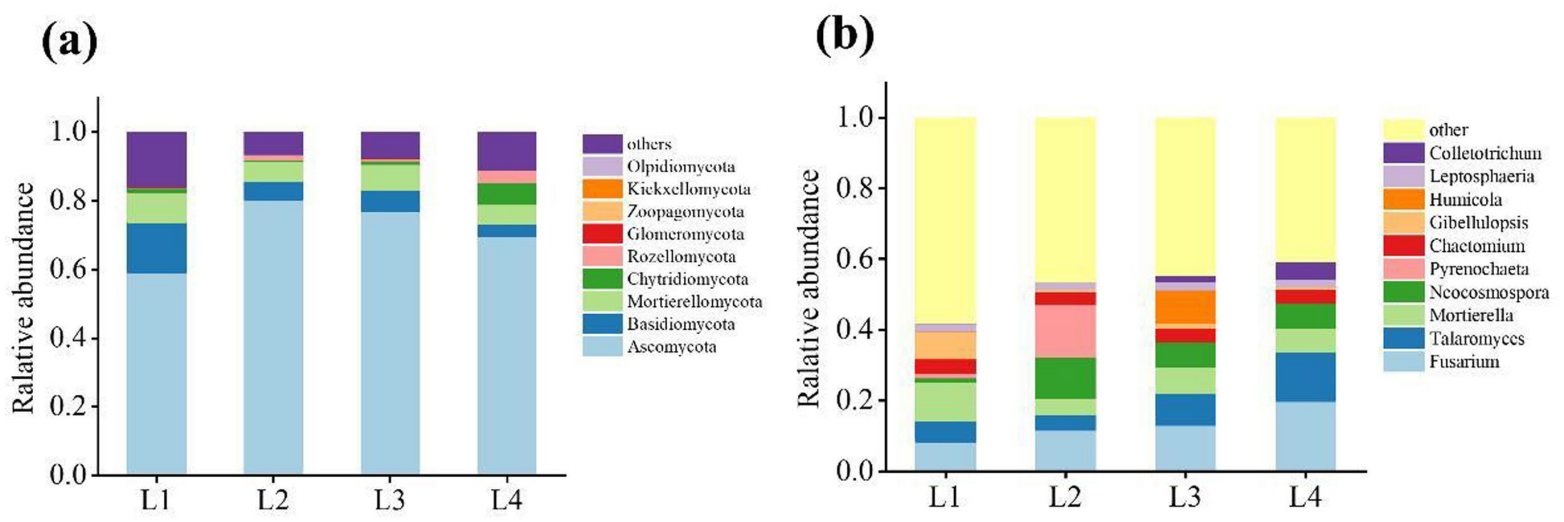
The distribution of dominant fungi in different continuous cropping years at phyla level **(a)** and genus level **(b)**.

The dominant fungi phylum was Ascomycota (59.03% ~ 80.26%), Basidiomycota (3.69% ~ 14.67%), and Mortierellomycota (5.61% ~ 8.73%) (Figure 5a). The relative abundance of Ascomycota increased significantly in L2, whereas the relative abundance of Basidiomycota and Mortierellomycota decreased after continuous cropping. Of the fungal genera whose relative abundance in the soil samples was greater than 1%, the relative abundance of Fusarium, Talaromyces, Mortierella, Neocosmospora, Chaetomium, Leptosphaeria differed significantly after different durations of continuous cropping (Figure 5b).

**Figure 6:**
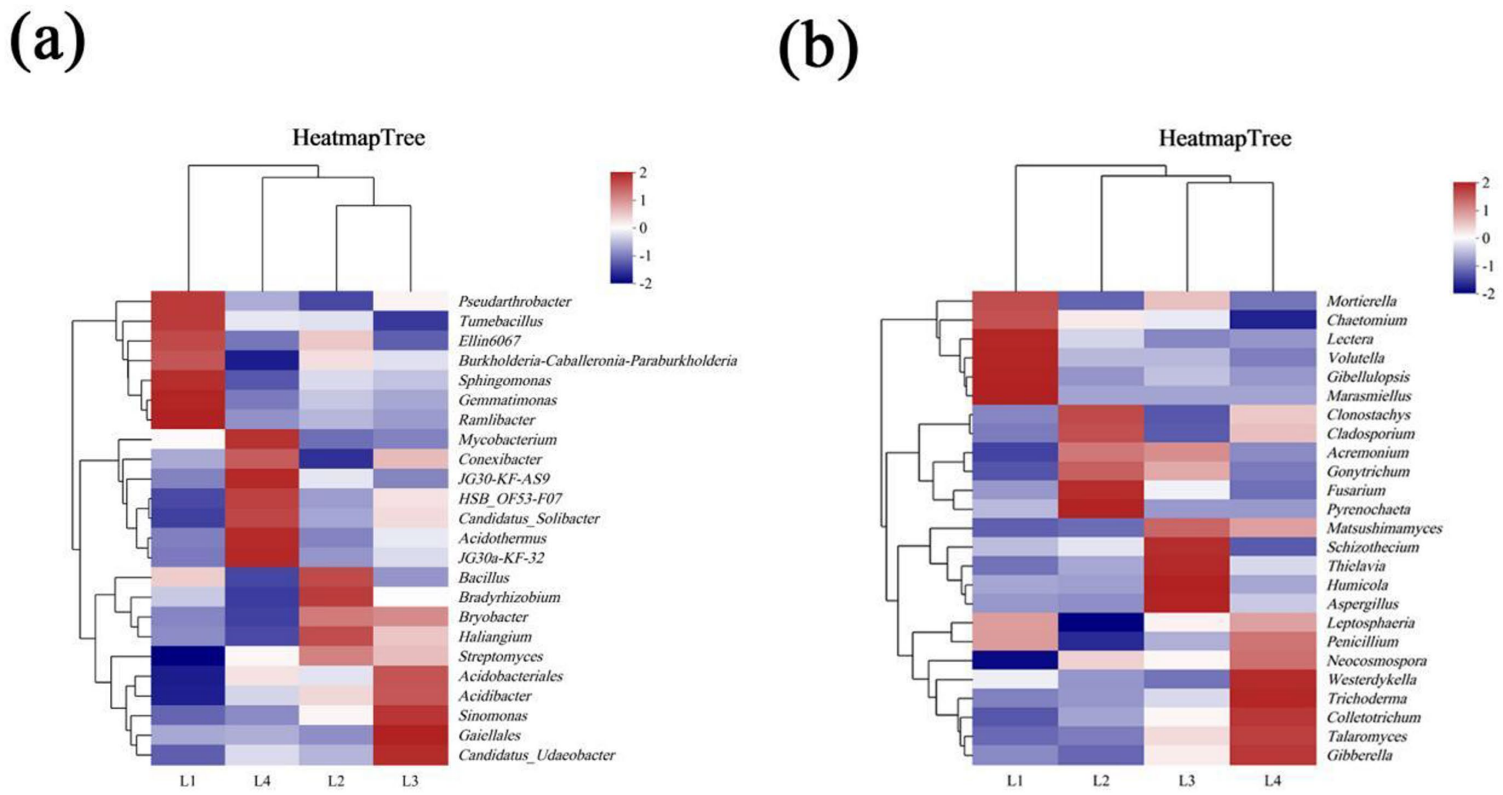
The distribution of dominant fungi in different continuous cropping years at phyla level **(a)** and genus level **(b)**.

The cluster heat map was used to analyze the differences in dominant bacterial genera in different years of continuous cropping. As shown in Figure 6a, there are certain differences in the types of dominant bacterial genera in different years. The dominant strains in L1 mainly included Sphingomonas (6.37%), Gemmatimonas (3.29%), Burkholderia-Caballeronia-Paraburkholderia (2.72%), Pseudarthrobacter (1.21%), Ramlibacter (1.21%), and Tumebacillusr (0.74%); The dominant bacterial genera in L2 were Bacillus (1.68%), Bradyrhizobium (1.45%), and Bryobacter (1.11%). The dominant bacteria in L3 were Acidobacteriales (6.34%), Gaiellales (4.59%), Candidatus_Solibacter (1.95%), and Candidatus_Udaeobacter (1.31%). The dominant strains in L4 mainly included JG30-KF-AS9 (8.50%), Acidothermus (6.24%), HSB_OF53-F07 (3.94%) and Conexibacter (2.42%).

The difference of dominant bacteria genera in different years of continuous cropping is shown in Figure 6b. There are certain differences in the types of dominant bacteria genera in different years. The dominant strains in L1 were mainly Mortierella (8.54%), Gibellulopsis (7.74%), Marasmiellus (2.43%), Volutella (1.65%), and Lectera (1.71%). The dominant strains in L2 were Fusarium (23.10%), Pyrenochaeta (14.89%), Clonostachys (2.60%), and Cladosporiumr (2.27%). The dominant strains in L3 included Humicola (9.50%), Aspergillus (2.36%), and Thielavia (2.02%). The dominant bacteria in L4 mainly included Talaromyces (13.20%), Colletotrichum (4.16%), Gibberella (2.46%), and Westerdykella (2.03%).

### 3.5 Principal component analysis (PCA)

**Figure 7:**
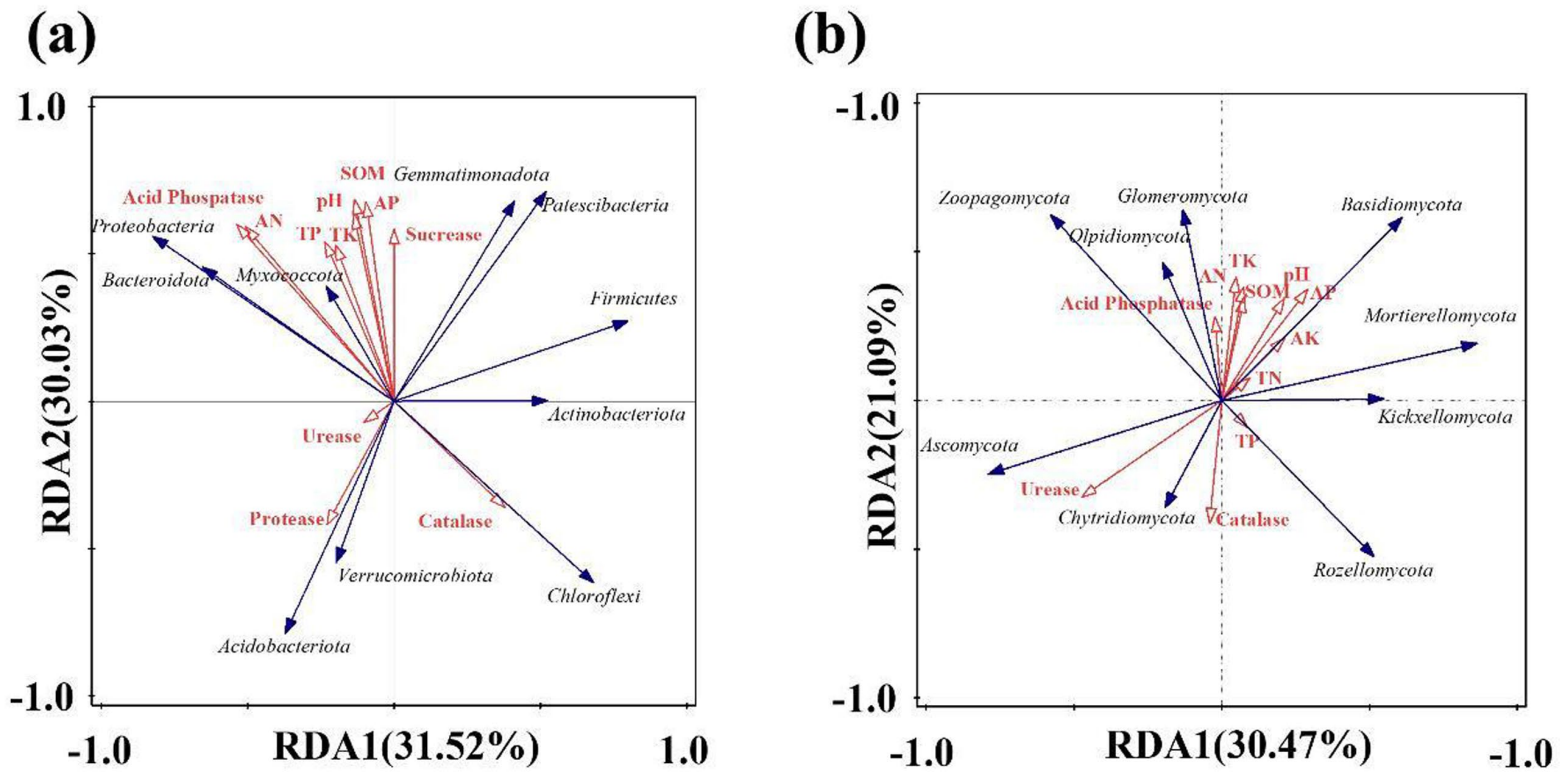
Redundancy analysis of soil environmental factors and microbial community in lily; **(a)** bacteria, **(b)** fungi.

Soil nutrients will affect the population structure and number of soil microorganisms, and the community structure and life activity of microorganisms will also affect the migration and transformation of soil nutrients. Redundancy analysis (RDA) was used to explore the relationship between soil nutrients and bacterial communities in the lily. As shown in Figure 7a, the variance interpretation rate for RDA analysis axis 1 is 31.52%, and the variance interpretation rate for axis 2 is 30.03%, resulting in a total interpretation rate of 61.55%. pH is negatively correlated with Acidobacteriota. Total phosphorus and potassium were positively correlated with Myxococcota, while total phosphorus, alkali-hydrolytic nitrogen, and acid phosphatase were positively correlated with Proteobacteria and Bacteroidota. Protease exhibits a significant positive correlation with Acidobacteriota and Verrucomicrobiota, whereas catalase shows a significant positive correlation with Chloroflexi and Actinobacteriota. As can be seen from Figure 7b, the variance explained by the RDA analysis axis 1 is 30.47%, that of RDA2 is 21.09%, and the total variance explained reaches 51.56%. Basidiomycota was positively correlated with the availablity of potassium and phosphorus. Acid phosphatase was positively correlated with Glomeromycota. Total phosphorus was positively correlated with Rozellomycota. Urease and catalase were positively correlated with Ascomycota and Chytridiomycota. According to the length of the arrow and the size of the angle, this study found that pH, SOM, alkali-hydrolytic nitrogen, and acid phosphatase were the main factors affecting the changes in bacteria, while urease and catalase were the main factors affecting the changes in fungi.

## 4 Discussion

Numerous studies have shown that after continuous cropping of lilies for 1 to 2 years, soil nutrients begin to decline significantly, while changes in the microbial community may not yet be apparent. After continuous cropping of lilies for 3 to 4 years, the soil microbial community undergoes significant succession. Soil-borne diseases typically emerge after 3 years of continuous cropping. If lilies are continuously cropped for more than 4 years, the soil may enter a “degradation stage.” This study analyzes the soil physicochemical characteristics and microbial community structure of lily continuous cropping over 4 years, encompassing the critical transition stage where continuous cropping obstacles shift from quantitative to qualitative changes.

### 4.1 Physicochemical degradation

The results of this study indicate that the pH decreased after continuous cropping. The average pH of lily soil was 5.07 after 4 years of continuous cropping. It is reported that the optimal soil pH for growing lilies of the genus Lilium is 5.5 to 6.5, but the soil becomes acidic after continuous planting. Soil pH is a crucial indicator of soil fertility, closely related to the formation, transformation, and availability of soil nutrients, and also affects the activity of soil microorganisms (Wang et al., 2019). Low pH may restrict root growth, reduce microbial activity, disrupt nutrient cycling, and thereby hinder plant nutrient uptake (Hong et al., 2023). Soil acidification may be caused by the accumulation of phenolic acid secretory substances in the root system of lilies during long-term continuous cropping or by excessive fertilization (Yan et al., 2020). According to RDA analysis, the change in pH value has a significantly higher impact on bacterial communities than on fungi, and pH value is a crucial factor affecting the structural changes of microorganisms. When Xu et al. (2021) studied the continuous cropping of Panax notoginseng, they found that pH value was the main environmental factor affecting the ratio of bacteria to fungi. Soil acidification resulted in a gradual increase in the proportion of fungi and a corresponding decrease in the proportion of bacteria. This is because fungi have higher acid tolerance than bacteria. When the soil pH value decreases, it is more conducive to the survival of fungi. This is also a key factor driving the shift from ‘bacterial-type’ to ‘fungal-type’ soil.

This study found that after two consecutive years of cultivation, the contents of SOM, TN, AN, TP, and AK in the soil decreased significantly. Soil SOM is closely related to plant nutrients and is an important source of nutrients such as N and P required for plant growth and development (Minasny et al., 2017). The higher the organic matter content, the stronger the microbial activity of the soil. It was found in this study that as the continuous cropping increased, the SOM content became lower, which would lead to a decrease in such microorganisms that degrade organic matter through catabolism. Nitrogen is the main component of amino acids and an important material basis for soil nutrients, influencing the growth and metabolism of plants (Nazir et al., 2016). The total nitrogen and alkaliolytic nitrogen contents followed the order L1 > L2 > L3 > L4. Phosphorus is a vital macronutrient involved in biological and physiological processes, including cell tissue formation, root development, seed production, and crop maturation. The content of available phosphorus decreased with the continuous cropping of lilies. The content of total phosphorus decreased 3 years before continuous cropping and increased 4 years after continuous cropping. This might be due to the transformation of microbial community functions. The differences in nitrogen and phosphorus may be related to the microorganisms that mediate their conversion.

The study found that key soil nutrient indicators declined significantly after 2 years of continuous planting. In actual production, intervention measures should be taken immediately after the second round of planting is completed, providing a time basis for formulating precise soil management plans. Secondly, the research revealed changes in indicators such as SOM, TN, AN, TP, and AK, and the fertilization strategy could be adjusted specifically based on these finding. These declining nutrient indicators can serve as early biomarkers for the continuous cropping obstacle, aiding in-depth mechanistic analysis.

Soil enzyme activity directly influences nutrient cycling and alters microbial composition. Additionally, it plays a key role in crop growth. In this study, both sucrose and acid phosphatase showed a downward trend. Sucrase, which catalyzes the hydrolysis of sucrose into glucose and fructose, is a key indicator of soil fertility and nutrient use efficiency. Sucrase is positively correlated with organic matter and available phosphorus, while acid phosphatase is significantly positively correlated with alkaline hydrolytic nitrogen and Proteobacteria. However, as the number of continuous cropping years increases, these environmental factors and Proteobacteria decline. The decline in sucrase and acid phosphatase activity impairs nutrient release, reduces soil fertility, and ultimately disrupts the balance of nutrients. Soil catalase decomposes hydrogen peroxide, phenols, and aldehydes, thereby mitigating accumulation of harmful substance. This study found that catalase activity initially decreased but later increased during continuous cropping, likely due to salt accumulation enhancing redox reactions. Therefore, the activity of catalase also shows an increasing trend with the increase of continuous cropping years. It may also be due to the influence of pH. Catalase exhibits a negative correlation with pH, and continuous cropping leads to soil acidification; therefore, the activity of catalase increases. Moreover, as shown in Figure 7, catalase is positively correlated with the Chloroflexi, Actinobacteriota, and Chytridiomycota. Since Actinobacteriota produce catalase, their trends align—both decrease initially and then rebound with prolonged cropping. This trend suggests that soil possesses a self-regulatory capacity during continuous cropping, as catalase activity rises to counteract the accumulation of harmful substances.

The changes in soil enzyme activities can serve as biological indicators of soil health, revealing the self-regulation mechanisms of continuous cropping soil systems and pointing the way for formulating precise soil management strategies.

### 4.2 Decline in beneficial microbial diversity

Soil microorganisms play a crucial role in maintaining the quality of the soil ecosystem and enhancing agricultural production (Khan et al., 2022). They not only participate in soil nutrient cycling, organic matter decomposition, and the transformation of elements like carbon and phosphorus, but also reflect the health status of the soil (Nannipieri et al., 2003). Bacteria are the most abundant and diverse group among soil microorganisms. They play a crucial role in agricultural ecosystems by participating in the circulation of soil nutrients, maintaining soil structure, and promoting plant growth (Li et al., 2014). The dominant bacterial phyla in this study are Proteobacteria, Actinobacteria, Chloroflexi, Acidobacteria, Firmicutes, Gemmatimonadetes, and Myxococcota. This is consistent with research on the continuous cultivation of sweet potatoes (Li et al., 2019) and bananas (Shen et al., 2018), although bacterial community composition may vary across plant species. Actinobacteria, Acidobacteria, and Proteobacteria are dominant in lilies and the aforementioned plants, suggesting their essential role in soil ecosystems. Fungi are key decomposers and play a vital role in maintaining the balance of the rhizosphere ecosystem, making them indispensable to the soil microbial community (Karthikeyan et al., 2008). In this study, Ascomycota, Basidiomycota, and Mortierellales were the dominant phyla, consistent with findings by Miao et al. (2016) on Panax notoginseng and Zheng et al. (2021) on apple fungal communities.

The Shannon and Simpson indices in this study showed that as continuous cropping years increased, bacterial diversity declined, while fungal diversity rose. As fungal abundance rose, the rhizosphere microbiota shifted toward a fungi-dominated composition. Soil acidification contributes to the dominance of this fungus. Similarly, Li et al. (2022) observed in lilies that fungal abundance rose progressively with longer continuous cropping.

Beneficial microorganisms such as Proteobacteria, Ascomycota, Mortierellomycota, Basidiomycota, Bacillus, Sphingomonas, Gemmatimonas, Mortierella, and Leptosphaeria are decreasing year by year, while the abundance of pathogenic bacteria such as Acidobacteria and Fusarium is increasing.

### 4.3 Pathogen accumulation

The main function of the Proteobacteria is to decompose organic matter and promote plant growth. Most nitrogen-fixing, ammonia-oxidizing, and denitrifying bacteria belong to the Proteobacteria (Wang et al., 2017). The reduction of Proteobacteria leads to a decline in biological nitrogen fixation capacity. Some Proteobacteria (such as Pseudomonas) participate in the nitrification/denitrification process, affecting the transformation of nitrogen forms. In this study, the abundance of Proteobacteria decreased with the extension of continuous cropping time, which is consistent with the research of Liu et al. (2014). Ascomycetes are saprophytic fungi in natural ecosystems. Ascomycota is the main force in the decomposition of lignin/cellulose. Its reduction will delay the decomposition of organic matter and lower soil fertility. In this study, the Ascomycota decreased with the increase in continuous cropping years, which is consistent with previous research results on the changes in the fungal community structure of cucumber continuous cropping (Sun et al., 2021). Mortierellomycota is a unique category in the kingdom of fungi, and most of its species are saprophytic fungi in soil. In this study, the abundance of the Mortierellomycota decreased from 14.67% in L1 to 3.69% with the increase of continuous cropping years. Basidiomycota is also one of the important decomposers of soil organic matter, promoting the circulation of soil nutrients and playing a crucial role in plant growth. At the same time, it has the function of decomposing wood and cellulose, and can promote the material circulation in the soil (Purahong et al., 2016; Zhao et al., 2013). After four consecutive years of cultivation, its abundance decreased by 74.91%. With the increase in continuous cropping years, the abundances of Bacillus and Sphingomonas gradually decreased. Sphingomonas is a common environmental microorganism that has the ability to fix nitrogen and nitrify in the soil nitrogen cycle, playing a role in resisting pathogen infection, thereby reducing the large-scale accumulation of toxic substances in the soil. In this study, the relative abundance of Sphingomonas in L4 was 68.49% lower than that in L1. Sphingomonas relies on complex carbon sources (such as polysaccharides), while the proportion of simple carbon sources (sugars in root secretions) in continuous cropping soil increases. The chemogenic substances released by continuous cropping may also inhibit its growth. Bacillus can produce toxins to inhibit the growth of harmful insects and pathogens in the soil (Hu et al., 2010). However, in this study, it was found that the abundance of Bacillus decreased from 13.80% in L1 to 11.66% in L4. Gemmatimonas is a genus of Gram-negative bacteria that is widely distributed and highly abundant in soil. Its most notable ecological function lies in its strong capacity for phosphorus solubilization. Through metabolic activity, these bacteria secrete organic acids that dissolve insoluble inorganic phosphorus compounds, such as calcium phosphate, converting them into forms that are plant- and microbial-available. This process significantly enhances phosphorus bioavailability, playing a vital role in facilitating the soil phosphorus cycle and supporting plant nutrient acquisition. However, this study observed a 53.37% decrease in the abundance of Gemmatimonas. Mortierella is a genus of filamentous fungi widely distributed in soil, plant root systems, and organic matter. It plays a vital role in soil ecosystems due to two main functions: first, as efficient decomposers, these fungi secrete a variety of enzymes that break down organic residues, facilitating the cycling of key elements such as carbon, nitrogen, and phosphorus; second, many species form symbiotic relationships with plant roots, enhancing water and nutrient uptake, producing plant hormones, and promoting root development. In this study, however, a decrease in the abundance of Mortierella was observed. Leptosphaeria is a genus of ascomycete fungi commonly found in soil and plant residues. As significant saprophytic organisms, they play a crucial role in decomposing recalcitrant plant materials—such as cellulose and lignin—thereby facilitating the breakdown of organic matter and carbon cycling. These fungi are essential contributors to soil nutrient dynamics and ecosystem sustainability. However, in this study, a decrease in the abundance of Leptosphaeria was observed.

The Acidobacteriota is greatly affected by pH. Acidobacteriota prefer acidic and low-nutrient environments. An increase in its abundance may reflect soil acidification or a lack of organic matter, further inhibiting the growth of beneficial microorganisms. Chu et al. (2010) found that the relative abundance of the Acidobacteriota phylum was significantly negatively correlated with soil pH, which is consistent with the results of this study. Therefore, in this study, the abundance of Acidobacteriota increased with the increase of continuous cropping years, as shown in Figure 4a, and the RDA analysis revealed a negative correlation between Acidobacteriota and pH, as shown in Figure 7a. In this study, the abundance of Fusarium in L4 increased by 57.73% compared to L1, which is consistent with the research result that continuous cropping significantly increased the fungal community of Fusarium in cucumber cultivation soil (Zhou and Wu, 2012). Continuous cropping crops constantly secrete the same carbon sources (such as phenolic acids and amino acids), and certain pathogenic Fusarium can efficiently utilize these substances for proliferation. Most strains within the Fusarium genus are pathogenic and can cause plant stem rot, root rot, scab, and other diseases. Therefore, long-term continuous cropping can lead to the occurrence of soil pests and diseases. As continuous cropping continues, beneficial microbes decline while pathogens accumulate, ultimately reducing lily yields. Neocosmospora is a genus of ascomycete fungi widely distributed in soil. It includes several notorious plant-pathogenic species capable of infecting the root systems of various economically important crops. These pathogens can cause symptoms such as root rot and wilting, leading to severe agricultural losses—exemplified by species known to induce stem base rot (Hao et al., 2021). In this study, an increase in the abundance of Neocosmospora was observed.

### 4.4 Soil adaptive mechanisms

Actinobacteriota first decreased and then increased with the increase of continuous cropping. Actinobacteriota are heterotrophs that produce chitinase, urease, and catalase, contributing to organic matter decomposition (Fuentes et al., 2014; Polti et al., 2014). At the same time, they produce antibiotics to inhibit various plant diseases, which is beneficial to plant growth. Firmicutes produce stress-resistant spores, which enhance survival under adverse conditions (Li et al., 2023). In this paper, the abundance of Firmicutes increases with the number of continuous cropping years, exhibiting a trend of first decreasing and then increasing, which, to a certain extent, enhances the soil’s ability to resist disease risks (Li et al., 2023). Wang et al. (2017) found that in continuous lily cultivation, the abundance of ammonia-oxidizing archaea initially decreased and then increased. This trend may be explained by the fact that, after continuous planting for a certain period of time, the soil will develop immunity, increasing the number of beneficial bacteria and enhancing its resistance to soil allelic substances, thus playing a self-regulating role (Wang et al., 2024).

These research results profoundly reveal the deteriorating trend of soil microbial community structure and its ecological impact during the continuous cropping of lilies, which is specifically manifested as a significant reduction in the abundance of beneficial microorganisms (such as Proteobacteria, Ascomycota, Bacillus, and Sphingomonas), while pathogenic bacteria (such as Fusarium and Acidobacterium) are significantly enriched. These microbial changes suggest that degradation of soil functions: weakened nutrient cycling capabilities such as nitrogen fixation and phosphorus dissolution, decreased inhibitory functions of pathogenic bacteria, and intensified soil acidification.

It should be emphasized that the inferences about the functions of beneficial and pathogenic microorganisms in this study are mainly based on the changes in taxonomic composition at the phylum and genus levels. However, the function and pathogenicity of microorganisms are usually determined by genes at the species or strain level. Therefore, the abundance changes at the gate and genus levels that we have observed may reflect the common changes among multiple species under it. The specific functional implications require more in-depth research for verification. Although this study has certain limitations, the research results can still provide data support for addressing the ongoing challenges of continuous cropping in lilies.

## 5 Conclusion

This study analyzed the changes in soil physicochemical properties, enzyme activities, and microbial community structure of four different continuous cropping years, namely L1, L2, L3, and L4, and mainly obtained the following conclusions:

1. After 2 years of continuous lily cultivation, soil nitrogen, phosphorus, potassium, and organic matter declined significantly, while catalase activity and abundances of Actinobacteria and Firmicutes rebounded in the third year. This indicates that the soil nutrients were no longer sufficient for lily growth after 2 years of continuous cultivation, and the self-regulation mechanism of certain microorganisms began to be activated, promoting the circulation of soil nutrients. Prolonged cropping increased soil sucrase and acid phosphatase activities but decreased catalase, protease, and urease activities. Sucrase correlated strongly with organic matter and available phosphorus, while acid phosphatase linked positively to alkali-hydrolyzable nitrogen. Catalase showed a significant negative correlation with pH.
2. Shannon and Simpson indices revealed a microbial shift from “bacteria type” to “fungi type,” disrupting community balance. With the increase of continuous planting years, the abundance of microorganisms such as Proteobacteria, Sphingomonas, Ascomycota, and Basidiomycota, which decompose organic matter and promote nutrient cycling, is decreasing, while the abundance of Acidobacteriota and Fusarium is increasing. The research found that the Actinobacteriota and Firmicutes exhibited a change, first decreasing and then increasing. The Actinobacteriota were correlated with catalase, and it was inferred that the soil might have a certain resistance to the continuous crop mechanism. Microorganisms and soil nutrients interact reciprocally: Acidobacteriota correlated with protease, Proteobacteria with alkali-hydrolyzable nitrogen, and Basidiomycota with available phosphorus and potassium. pH, SOM, alkalohydrolytic nitrogen, and acid phosphatase are the primary factors influencing bacterial changes, while urease and catalase are the key factors affecting fungal changes.

The research results are of great value in overcoming the obstacles to continuous lily cultivation, optimizing soil management and improving the sustainability of planting. By analyzing the changes in soil physical and chemical properties, enzyme activities, and microbial communities under different continuous cropping years, the key mechanisms of soil nutrient decline and microbial imbalance caused by continuous cropping were revealed, providing a scientific basis for formulating targeted measures, which are conducive to achieving the sustainable development of the lily industry. The research results can guide soil management strategies to alleviate continuous cropping obstacles, including the inoculation of compound functional microbial agents, the application of organic conditioners to regulate soil physical and chemical properties, the optimization of crop rotation systems to break the transmission chain of pathogenic bacteria, and the screening of lily varieties that interact well with beneficial microorganisms. Through precise soil management, the ecological functions of the soil can be effectively restored, obstacles to continuous cropping can be alleviated, and sustainable high-yield cultivation of lilies can be achieved.

## Data Availability

The datasets presented in this study can be found in online repositories. The names of the repository/repositories and accession number(s) can be found in the article/supplementary material.

## Footnotes

^1^ http://huttenhower.sph.harvard.edu/galaxy/root?tool_id=lefse_upload
